# Effect of Dehydration on the Resilient Modulus of Biopolymer-Treated Sandy Soil for Pavement Construction

**DOI:** 10.3390/polym17202738

**Published:** 2025-10-13

**Authors:** Ahmed M. Al-Mahbashi, Abdullah Almajed

**Affiliations:** Bugshan Research Chair in Expansive Soils, Department of Civil Engineering, College of Engineering, King Saud University, Riyadh 11421, Saudi Arabia; alabduallah@ksu.edu.sa

**Keywords:** biopolymer, sand, resilient modulus, dehydration, suction, subgrade materials, soil water retention curve

## Abstract

Biopolymers have recently been introduced as eco-friendly alternatives to other chemical cementitious additives for sandy soil stabilization, especially in pavement construction. The resilient modulus (M_R_) is a key metric considered in the mechanistic design of pavement layers that ensures a safe and economic design based on guaranteed accurate values. This study investigated the effects of dehydration on the M_R_ of biopolymer-treated sand. Prepared specimens were subjected to two different curing conditions. The first set underwent closed-system curing (CSC) for periods of 7, 14, and 28 days. The second set of specimens was cured at different levels of suction by controlling relative humidity (RH) using different salt solutions (0.27, 1.0, 9.7, 21.0, 54.6, 113.7, and 294 MPa), referred to as dehydration curing (DC). The soil water retention curve (SWRC) was measured over the entire suction range to evaluate the dehydration curing and to link the results of suction levels and dehydration regime. M_R_ tests were conducted on both sets of specimens using a dynamic triaxial system to simulate different confining, traffic, and dynamic stresses. The results showed a significant increase in M_R_ (i.e., up to eight times) for specimens cured under DC conditions that was proportional to the suction level across different zones of the SWRC. Scanning electron microscopy revealed a phase change from hydrogel to film, which enhanced cementation and bonding between particles. in addition, CSC treatment resulted in a 10–30% reduction in M_R_. A new regression model is proposed to predict the M_R_ of biopolymer-treated sand as a function of confining stresses, dynamic stresses, and suction. These findings will assist pavement engineers and designers in achieving safe, sustainable, and economic designs.

## 1. Introduction

Sandy soils are widely distributed across many regions worldwide. These materials are cohesionless and inherently weak in sustaining external stresses. In large infrastructural projects such as highways and airports, where sandy soils are commonly used for pavement design, special mitigation methods are therefore required to improve their engineering properties. Recently, several studies have explored the use of biopolymers as environmentally friendly soil stabilizers in highways and other infrastructure projects, offering alternatives to chemical additives that can harm the environment through carbon emissions and by generating harmful products that affect underground soil and water (i.e., [1,2,3]). Several of these studies have specifically examined sandy soils (i.e., [4,5,6,7,8,9,10,11]), whereas a smaller number have focused on fine-grained or expansive soils [12,13,14,15,16]. The primary findings demonstrate that biopolymer treatment significantly improves the mechanical behavior of sand, including unconfined compressive strength, cohesion, and stiffness. The aforementioned improvements achieved by biopolymer treatment are attributed to several key reactions and mechanisms. Biopolymers create cross-links between soil particles through adhesive bonding, forming a cementitious gel that fills the pores in the soil structure [17]. Moreover, biopolymer–soil interactions involve multiple mechanisms: biopolymers occupy pore space, change soil pore volume, and retain water and thus their swelling behavior can induce soil volume changes that are constrained by soil particles [18]. In addition, the hydrophilic nature of biopolymers influences swelling and compressibility through charge exchange and related chemical reactions.

Few studies have focused on the behavior of treated soil under cyclic or repeated loading. Ni et al. [19] investigated the effect of biopolymer (1–5%) treatment on fatigue resistance (i.e., number of cycles to failure) for Shanghai clay. Both constant and stepped amplitudes of loading were applied. The results indicated that regardless of the applied amplitude, the biopolymer significantly enhanced the soil bearing capacity and fatigue life.

However, the use of these biopolymers as stabilizers depends on the curing conditions under different circumstances, which are rarely addressed and require further investigation. Lemboye and Almajed [20] investigated the effects of different biopolymers (acacia, sodium alginate, and pectin) on the unconfined compressive strength of dune sand. The study was conducted under different curing conditions with respect to temperature (25–110 °C), similarly to previous studies, and the biopolymers in general enhanced shear strength. However, the effect of temperature showed a double trend. Sand treated with sodium alginate exhibited a slight increase in strength with curing temperature up to 90 °C, followed by a decrease due to decomposition of cementitious compounds at higher temperatures. Further studies conducted by Vydehi and Moghal [21] evaluated the possible effects of biopolymers (xanthan gum and guar gum) on the strength and compressibility of low-plastic soils. That study considered the effects of natural and controlled curing. In the natural curing method, the specimens were exposed to open air or atmospheric temperature for different periods, and the controlled curing specimens were stored in a dissector with ambient temperature and relative humidity (RH) varying daily (RH: 20–95%). The results of the specimens treated at atmospheric natural temperature showed a significant increase in USC values compared to specimens treated under controlled temperatures. This enhancement was attributed to the fast dehydration of the specimens treated under natural conditions, which led to the hardening of the biopolymer gel component. However, the study was unable to establish a link between the hydration process and the level of suction for the tested specimens, which could support modeling and designing approaches that consider field moisture variation and corresponding suction variations.

The resilient modulus (M_R_), a measure of soil stiffness, is considered a fundamental input parameter for the structural design and analysis of flexible pavements according to the National Cooperative Highway Research Program (NCHRP) [22]. Accurate determination of M_R_ is essential for the mechanistic design and analysis of pavement layers. In several cases, subgrade layers require improvement to enhance the M_R_ values for safe and economic design. Previous studies that used cementitious additives such as lime hydroxide or cement have reported increases in M_R_ exceeding threefold, with curing time playing a significant role in these improvements [23,24,25,26,27,28,29]. In recent years, owing to rapid climatic changes and their consequences on the environment, the focus has shifted to the use of environmentally friendly biopolymers to enhance soil properties and stiffness response under movable traffic loads. To the best of our knowledge, very few studies have investigated the possible effects of biopolymer treatment on the M_R_ of subgrades (i.e., [30,31]). Georgees et al. [30] evaluated the effect of polyacrylamide polymer (i.e., 0.002% dry weight) treatment on the resilience characteristics of three granular soils as pavement materials: poorly graded sand (SP-SM), sandy clay (CL), and clayey sand (SC). The specimens were prepared under initial conditions of optimum moisture content and maximum dry density according to standard compaction curves. Before commencing the experimental tests of M_R_, the specimens were dehydrated to 50% of their optimum moisture content. The increases in the resilient modulus compared to the untreated soil were found to be approximately 35%, 55.8%, and 8.8% for each soil, respectively. The study justified this different response to the soil type and the adsorption of polymer particles onto the surface of the specimens, which in turn affected the bonding of particles and consequently the strengthening. The overall improvement enhanced the empirical design and reduced the required thickness of the pavement layer. However, the study did not consider higher dehydration rates and did not explore the full range of suction.

Similarly, Kumar et al. [31] conducted a series of laboratory tests to evaluate the effect of an emulsion polymer (synthetic vinyl copolymer family) on the M_R_ of treated silty sand soil. Three dosages of polymer emulsions were considered, 4%, 5%, and 6%, and three curing periods were considered, 6 h, 3 days, and 7 days, under a constant relative humidity of 50%. The specimens that were allowed to dehydrate for 7 days exhibited an increase in M_R_ by a factor of approximately 6. Subsequently, no further plausible improvement in the M_R_ values was observed with an increase in the curing period up to 14 days.

Another critical factor is the dehydration and variation in moisture content, which is linked to the variation in soil suction. Moisture variations may occur during construction before sealing the constructed materials with upper pavement layers, when dehydration can reach as high as 60%. Fluctuations in the groundwater table can also contribute significantly to dehydration and consequent fluctuations in soil suction [32,33]. Evaporation through propagated cracks may contribute to dehydration. Research on the M_R_ characteristics of biopolymer-treated sand remains limited, particularly with respect to the dehydration process over its lifetime and its relationship to different suction levels across the soil water retention curve (SWRC). This study examined the effect of dehydration on the M_R_ of biopolymer-treated sandy soils. Two distinct conditions were considered for curing the prepared specimens under a suction-controlled environment: closed-system curing (CSC) and dehydration curing (DC). The CSC specimens were cured for 7, 14, and 28 days. By controlling the relative humidity (RH) with various salt solutions, we cured the DC specimens under varying suction levels (0.27, 1.0, 9.7, 21.0, 54.6, 113.7, and 294 MPa). To assess the dehydration curing process and correlate results with the suction levels and dehydration zones, the SWRC was evaluated in the laboratory over the entire suction range. For both curing conditions, M_R_ tests were conducted using a triaxial dynamic system to simulate various confining stresses (CP) and traffic or dynamic stresses.

## 2. Materials and Methods

**Figure 1:**
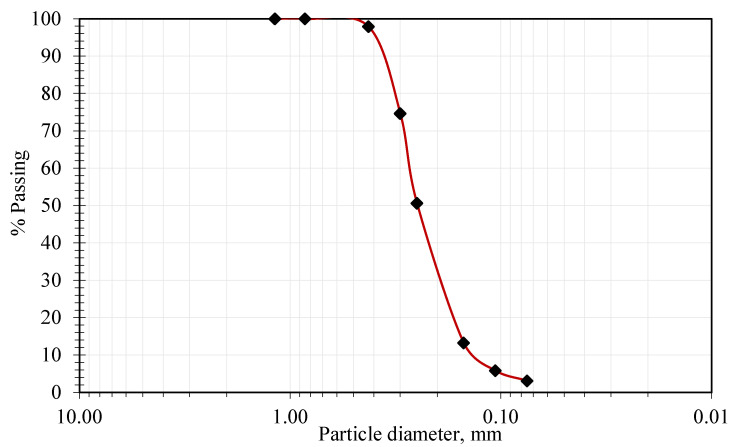
Particle size distribution of the soil used.

**Figure 2:**
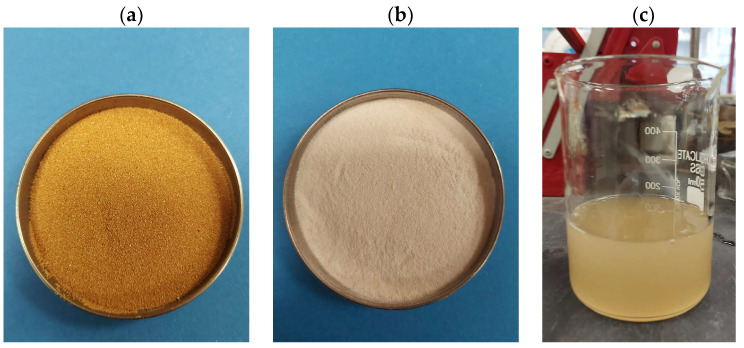
Visual representations: (**a**) physical image of the reddish sand used in this study, (**b**) sodium alginate powder, and (**c**) ionic gel of sodium alginate formed in the presence of divalent or trivalent cations.

Sandy soils and dunes cover vast areas of Saudi Arabia in different regions: the northern sand source, the central zone, and the southern zone. The characteristics of these sands differ according to their source and mineral composition. The development of these areas surrounding urban zones typically requires full integration into a network of highways and roads. Most of these sandy soils consist mainly of quartz and silica, with some fine materials introduced among the sand particles because of floods or dust storms [34]. In several cases, fine materials have been added to control the hydraulic behavior and improve the bonding between the particles of these materials. An example of these additions is expansive clay, which is used in liners and protective layers for waste disposal [35,36]. The sand used in this study was sourced locally from the Althumamah area near Riyadh. The particle size distribution curve shown in Figure 1. The curve demonstrates a range of particle sizes between 0.5 and 0.1 mm. This sand contained fine materials of high plastic soil (i.e., expansive soil), with 3.2% passing the #200 equal sieve. The uniformity coefficient (Cu) was 1.957 and the curvature coefficient 0.989. The soil was classified as poorly graded sand (SP-SM) according to the Unified Soil Classification System ASTM D2487 [37]. The sand was reddish in color, and a physical image of this sand shown in Figure 2a.

Sodium alginate (SA) was selected in this study due to its film-forming, thickening, and gelling properties, as well as its accessibility, sustainability, and ease of usage. This biopolymer SA is a water-soluble linear polysaccharide derived from alginic acid that consists of α-l-guluronic (G) and 1,4-β-d-mannuronic (M) acids. Notably, SA, a component of the cell walls of marine brown algae, contains 30–60% alginic acid. The color of SA is yellow to light brown, as shown in Figure 2b, and its viscosity ranges within 25–39 cps. In the presence of divalent and trivalent metal cations, SA produces an ionic gel (Figure 2c), which is beneficial for soil treatment [38,39,40]. The SA biopolymer dose utilized in this investigation was 3%, which is considered an optimum value for effectively enhancing the strength and hydromechanical characteristics of soils (i.e., [11,19,31,41]).

## 3. Experimental Program

### 3.1. Specimen Preparation

The specimens were prepared under the optimum conditions obtained from the compaction curve, including maximum dry unit weight and optimum water content, as determined using the standard compaction test [42]. As mentioned earlier, a 3% SA biopolymer dosage was considered, which is within the most common practical and optimum range. The maximum dry density obtained from the compaction curve was 18 KN/m^3^, and the optimum moisture content was 13.6%. The biopolymer was amended with distilled water, and the optimum moisture content was added to the dry soil and mixed thoroughly. This method is widely used to ensure homogeneous mixes, guaranteeing even distribution of biopolymers throughout the sand particles and improving treatment consistency [43]. Mellowing of the mixture is an important process to ensure homogeneity, and a minimum of 24 h was allowed for this purpose. For the SWRC measurements, the specimens were statically compacted to a height of 20 mm and a diameter of 50 mm. The specimens used for the M_R_ tests measured 50 mm in diameter and 100 mm in height. These specimens were statically compacted to the target dimensions using a double plunger mold, as specified in BS EN 13286-53 [44].

### 3.2. Determination of SWRC

The relationship between soil suction and water content is described by the SWRC, which has wide applications in designing or modeling unsaturated soils at a specific level of suction or corresponding water content. In this study, the SWRC was used to define and correlate the dehydration potential to a specific level of suction, thereby approximating the variations in soil suction occurring in the field during drying or dewatering of groundwater in the long term.

The axis translation technique is the most common technique used to define the SWRC up to a suction value of 2000 kPa, as outlined in ASTM D6836 [45]. A pressure plate apparatus was used in this study for this purpose. Further details about this device and its main components are documented in the literature [46,47,48]. This technique imposes suction by applying air pressure to a saturated specimen inside an isolated chamber. The specimen was in complete contact with a ceramic disk with a proper high air-entry value to separate the air and water phases. Suction was imposed in increments of 5, 10, 20, 50, 100, 200, 400, 800, and 1500 kPa, and water was expelled from the soil specimen and collected inside the upper chamber beneath the ceramic disk, which assisted in monitoring the outflow of water and evaluating the equilibrium state. Equilibrium was assumed when no further water outflow was observed overnight for each increment, and the corresponding water content for each suction level was determined.

For further measurements of soil suction greater than 1500 kPa, the vapor equilibrium technique was utilized following the designation of ASTM E 104 [49]. The specimens were maintained in a non-contact condition above af saturated salt solution with a known relative humidity (suction). The entire setup was placed inside an airtight box, and the specimen was allowed to reach equilibrium under suction. During this process, water molecules in the vapor phase migrate to or from the soil pores. The weight of the specimen was monitored and recorded at short intervals of 2–3 days. Equilibrium was assumed once no further changes were observed in successive weight measurements. At equilibrium, the relationship between the relative humidity and total suction was computed using the Kelvin equation [50], as shown in Equation (1). Similarly to previous steps, the final water content corresponding to each suction level was determined. The entire SWRC profile was depicted between the degree of saturation and soil suction.

(1) $$\psi =\frac{RT\rho_{w}}{w}ln\left[\frac{1}{RH}\right]$$

where:

*ψ*: soil suction (kPa)

*R*: molar gas constant (8.314462 J/(mol K))

*T*: absolute temperature (K)

*w*: molecular mass of water vapor (18.016 g/mol)

*ρ_w_*: mass density of water (Kg/m^3^)

*RH*: relative humidity

### 3.3. Curing Process

To highlight the side effects of dehydration on the MR values of the tested materials, two curing methods were considered, as mentioned earlier: closed-system curing (i.e., CSC) and dehydration curing (i.e., DC). The following sections describe both the curing methods in detail.

#### 3.3.1. Closed-System Curing

In CSC, specimens were wrapped in plastic films and cured in a constant environment at a temperature of 23 ± 2 °C and relative humidity exceeding 95% for 7, 14, and 28 days. The moisture content was maintained at a constant level, and no dehydration was allowed.

#### 3.3.2. Dehydration Curing

In DC, curing was conducted under different levels of dehydration defined by the corresponding levels of suction and achieved by controlling RH using different salt solutions. In design and modeling, linking soil behavior in its unsaturated state to specific suction levels is a common approach, and the SWRC plays a crucial role in defining this state. Establishing such a relationship allows the selection of appropriate control points for soil behavior across the entire suction range, ensuring safe and economical designs.

The points selected in this study were represented on the SWRC, covering different stages up to the residual condition. Different saturated salt solutions were used to ensure dehydration under specific rate and suction conditions. Each solution had a known RH and corresponding suction level. In addition, a NaCl solution of a known concentration of NaCl was prepared for use in accordance with ASTM D 5298 [51]. Table 1 summarizes the set of saturated salts and salt solutions used in this study, along with their corresponding nominal suction and relative humidity values.

**Figure 3:**
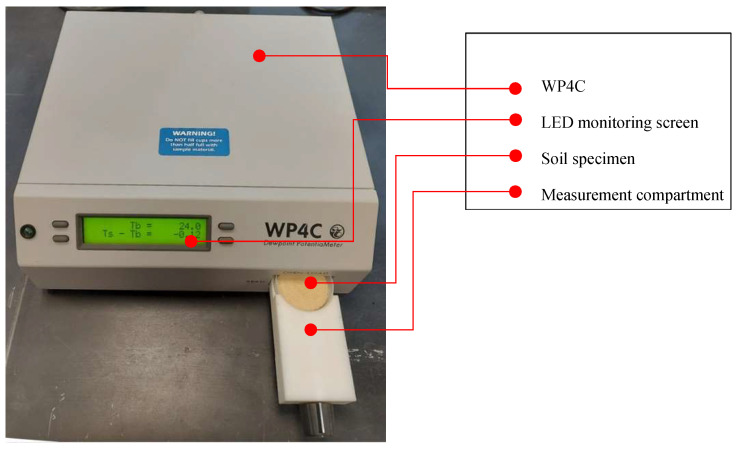
WP4C potentiometer.

These salt solutions were used to dehydrate specimens under constant relative humidity and to achieve known suction levels in the range of 0.05–114 MPa. To cover the entire suction range, two additional specimens were prepared: one in the compaction state and another dried in open air to reach the maximum residual suction. The nominal values of suction imposed by each RH (saturated salt and salt solution) were examined after equilibration using a WP4C potentiometer developed by Decagon^®^ (Pullman, WA, USA) [45], as presented in Figure 3.

**Figure 4:**
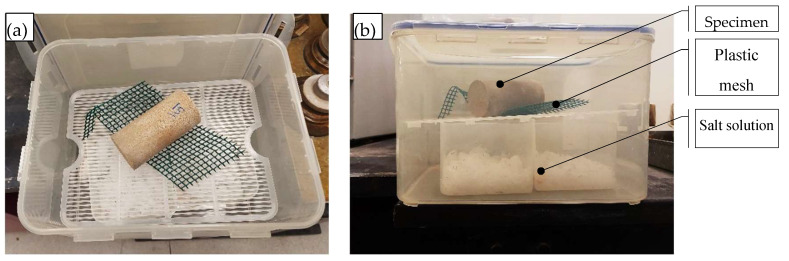
Dehydration under different RH and suction: (**a**) specimen suspended above the salt solution; (**b**) test setup inside an airtight box.

Several isolated boxes were prepared for each solution. Each specimen was suspended in non-contact with the salt solution using a plastic mesh, as shown in Figure 4a, and the entire setup was placed inside an airtight box (Figure 4b) to achieve equilibrium.

**Figure 5:**
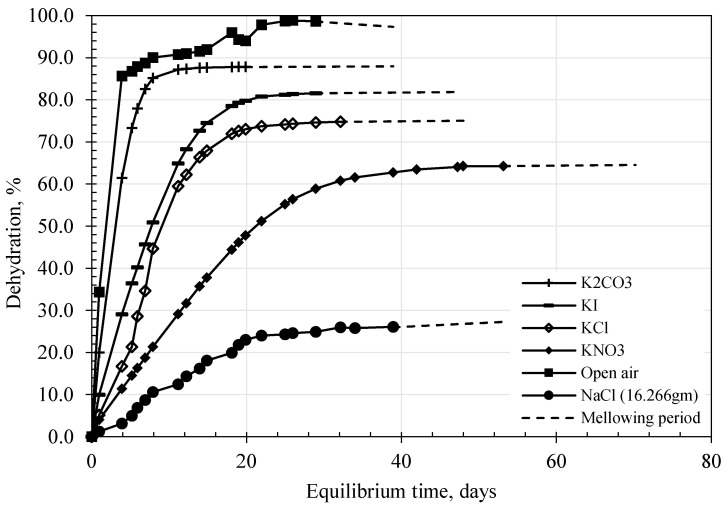
Dehydration process and equilibrium under different RH (suction).

**Figure 6:**
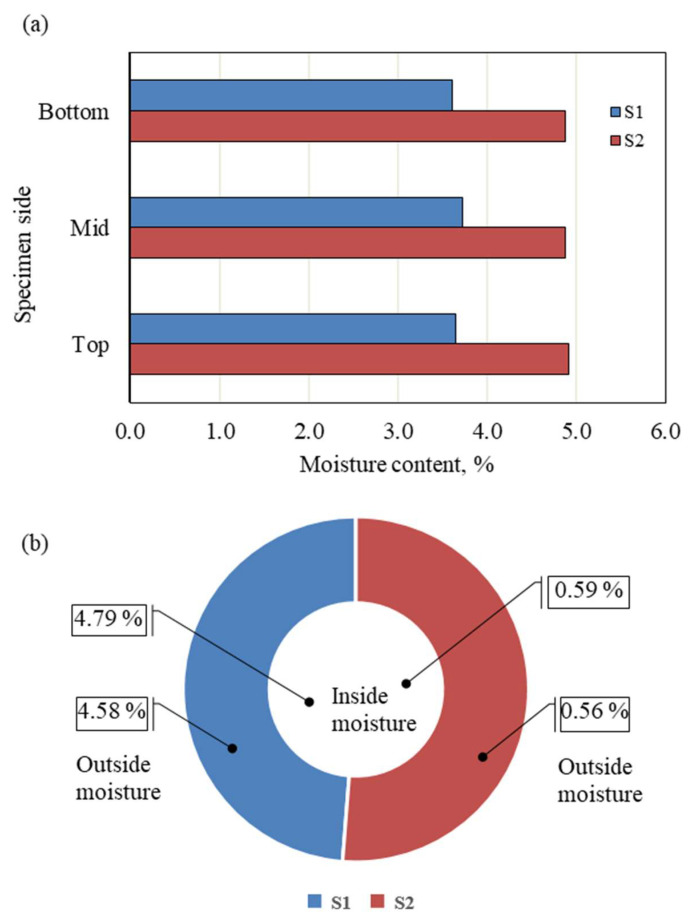
Variation in water after DC: (**a**) longitudinal; (**b**) inside and outside of the specimen.

During the equilibrium period, the specimens were periodically weighed and the relationship between time and dehydration calculated, as shown in Figure 5. Equilibrium was assumed when no further changes in the dehydration rate were observed at each suction level. After equilibration, the specimens were wrapped tightly in plastic film and allowed to mellow for at least 10 days before testing. This was a precautionary measure to ensure the uniform distribution of water along the specimen. Drying or dehydration was not allowed during this period, and the dashed line in Figure 5 represents the mellowing period. Duplicate specimens were used in the study. The specimens were sliced into top, middle, and bottom layers. The moisture content of each layer and the water content inside and outside the specimens were determined, and the results are plotted in Figure 6a,b. The variation from the average value along the specimens ranged from 0.07% to 1.9%, while the variation between inside and outside values of the tested specimens was less than 5%.

To identify microstructural changes induced by dehydration, the specimens were scanned using field-emission scanning electron microscopy (FE-SEM) at 5–10 KV.

### 3.4. M_R_ Testing

**Figure 7:**
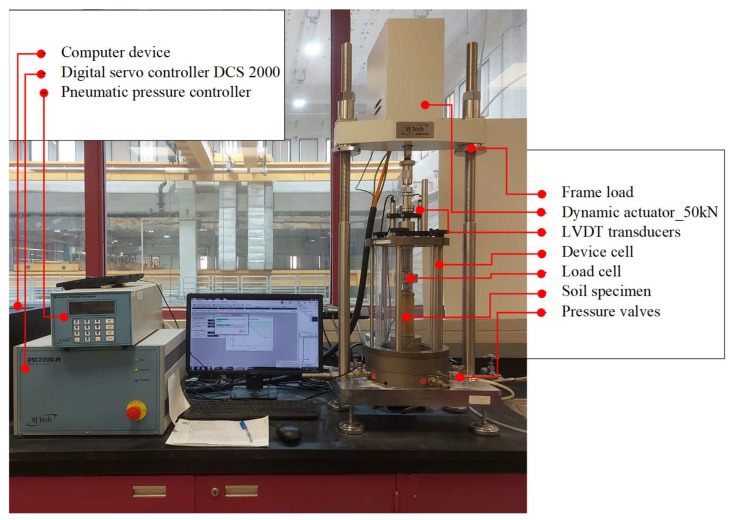
Dynamic triaxial system utilized for M_R_ testing.

The resilient modulus (i.e., M_R_) defines the relationship between the stress and strain responses of soil under cyclic loading conditions. This loading was stimulated by the expected repeated loads of traffic: several axial stresses (AS) were applied under different confining stresses of 13.8 kPa, 27.8 kPa, and 41.4 kPa as per AASHTO T-307 [52]. A dynamic triaxial system provided by VJ Tech^®^ (Reading, UK) was used to conduct the tests. The entire system is illustrated in Figure 7, where the main components are also highlighted. The setup was connected and operated by appropriate software connected to a computer. The tests were conducted in the laboratory on tread specimens after curing. The specimen was encased in a rubber membrane inside a device cell to facilitate the application of the confining stress. The test was conducted using a triaxial dynamic system by an automated, closed-loop servo electromechanical loading system (VJ Tech^®^, Reading, UK) (Figure 7). The shape of the applied cycle was haversine with a duration of 0.1 s and a rest period of 0.9 s. As previously mentioned, the actuator was applied with successive cycles at this rate, and a pneumatic air pressure controller was used to apply the confining stresses. An attached load cell and linear variable differential transformers (LVDTs) were used to record the applied stresses on the specimen and the corresponding deformation. The deformation was considered the average value obtained from the two transducers.

The tests comprised 15 sequences of load stages conducted at 3 confining pressures (13.8, 27.6, and 41.4 kPa) and 5 distinct axial stresses (13.8, 27.6, 41.4, 55.2, and 68.9 kPa), in addition to the initial conditioning stage (sequence 0). Specifically, the conditioning phase (sequence 0) was performed by applying a minimum of 500 repetitions with a haversine-shaped load equivalent to a maximum axial stress (MAS) of 27.6 kPa and a confining pressure of 41.4 kPa. This phase was necessary to improve the seating conditions and minimize the imperfect contact between the sample cap and test specimen. Subsequently, 15 sequences of 100 repetitions each were applied to the specimens using different levels of confinement and axial stresses.

## 4. Results and Discussion

### 4.1. SWRC and Selected Points for DC Treatment

**Figure 8:**
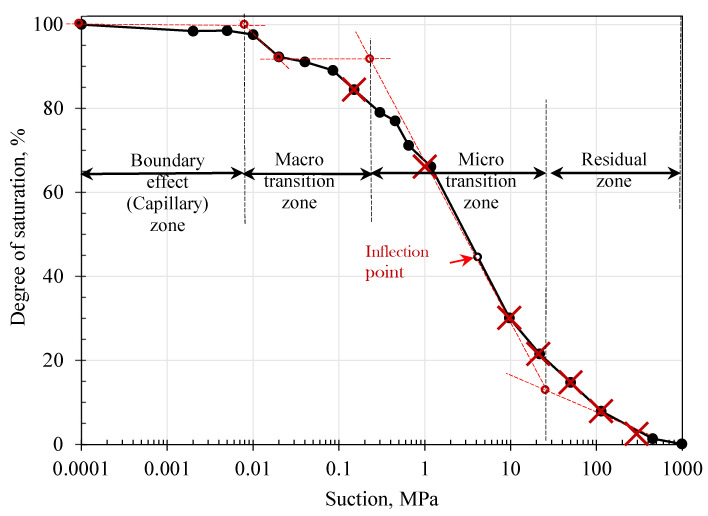
SWRC and selected points for DC treatment.

Figure 8 depicts the obtained results for SWRC, with the data presented in terms of degree of saturation (S%) versus suction (ψ). The curve exhibits a bimodal shape. This behavior is commonly encountered in sandy soils with fine particles or poorly graded soils, and the compensation between these two series of particles provides two levels of micropores and macropores (i.e., [53,54,55]). The bimodal SWRC is characterized by two identifiable air entry values (AEV_1_ and AEV_2_), and two residual points (S_r1_, ψ_r1_; S_r2_, ψ_r2_), as shown in Figure 8. In these soils, the second residual state is the most relevant. The AEV is defined as the value of suction where the dominant large pores start to desaturate, and the residual point is the limit where no further changes take place during further dehydration. These points function as control points and divide the SWRC into three zones: capillary, desaturation, and residual. For a bimodal SWRC, the transition zone can be divided into macro- and micro-transition zones [54,56]. The characterization of these zones is governed by the water content inside the soil pores and varies with the introduction of the dehydration process along the suction range. The boundary effect zone was controlled by capillary force, because most of the soil pores were filled with water. As dehydration continues, the water starts to discharge from soil pores, and adsorption forces govern this zone and the residual zone, where only the water regime strongly attached to soil particles remains. The capillary zone of this soil was bounded by AEV_1_ (8 kPa), and most of the soil remained saturated. The AEV_2_ was estimated to be approximately 250 kPa, and the residual zone started at 23 MPa and 11%.

The behavior of soil under any dehydration level can be better interpreted in relation to these zones, and it is essential to consider the modeling and design parameters in the unsaturated state. In Figure 8, the red crosses refer to the selected points for the DC in different zones. Hereafter, the M_R_ test point is interpreted and linked to these zones and their levels of suction.

### 4.2. M_R_ Result Selection and Statistical Variation

**Figure 9:**
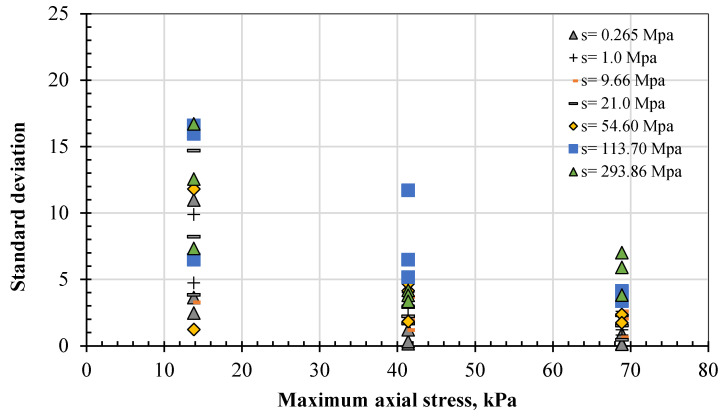
Variety of standard deviations for M_R_ tests under DH curing.

This test involved a large number of repetitions, which enhances reliability: 15 sequences of the load stage were conducted in addition to the initial conditioning stage (sequence 0). The initial conditional sequence comprised 500 cycles, and each of the other sequences comprised 100 cycles. The values presented in the results represent the average of the last five cycles for each sequence, as advised by AASHTO T 307 [52]. The standard deviations associated with these values for the specimens tested under DH curing (i.e., 7 specimens) are shown in Figure 9. Except for the first or initial states at the lower maximum axial stress, the standard deviation varied from a low value of 0.142 and was mostly limited to 5. The maximum and minimum values were 45 and 421, respectively, and the median value for this range was 252.

### 4.3. Effect of Closed-System Curing (CSC) on the M_R_

**Figure 10:**
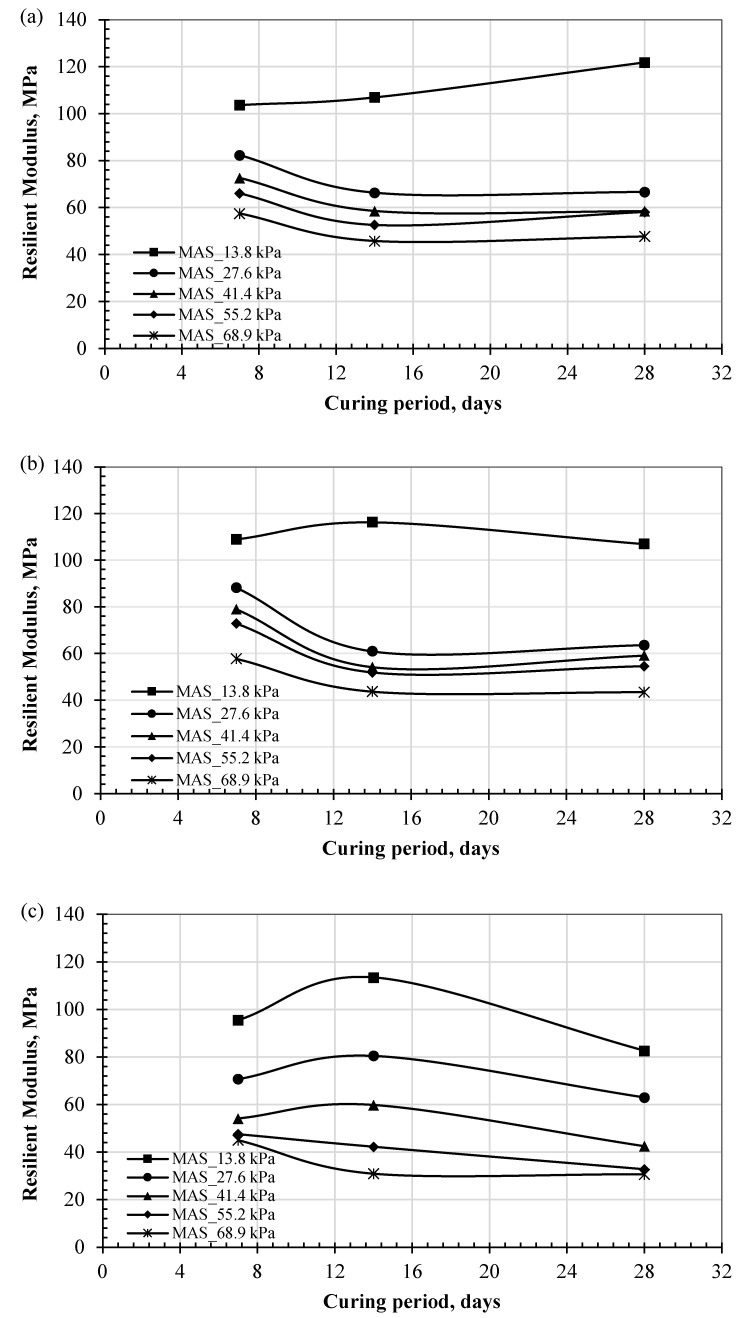
Effect of CSC on M_R_ results under confining pressures of (**a**) 13.8 kPa, (**b**) 27.6 kPa, and (**c**) 41.4 kPa.

This section presents the experimental results for the M_R_ values of the specimens subjected to CSC. The results depict the variation in M_R_ with different curing periods (i.e., 7, 14, and 28 days), as shown in Figure 10a–c for the test conditions for confining pressures of 13.8, 27.6, and 41.4 kPa, respectively.

In general, the M_R_ values decreased with an increase in the curing time from 7 to 14 days, and no further significant changes were observed. One exception occurred at low axial stress (13.8 kPa), where a modest increase of up to 17% was observed. This variation in the M_R_ with the curing period and axial stress can be attributed to the strain-softening behavior, where the presence of water weakened the bonding between particles. At lower axial or deviator stresses, the applied load was insufficient to activate strain-softening behavior. However, with increased axial stress, softening effects developed, resulting in a reduced M_R_ [57,58].

The reduction in M_R_ values observed with curing time up to 14 days can also be attributed to further interactions between the biopolymer and soil particles. These include mellowing of gelatinous compounds in the biopolymer gel or chemical ionic reaction, which enhanced the strain-softening behavior. In addition, the biopolymer–soil interaction involved occupying pores, altering volumes, and retaining water, which in turn increased compressibility [18] and reduced resilience properties. This interaction eventually stabilized, resulting in no further changes in the M_R_ values.

The general trend indicates a decrease in M_R_ values apart from the modest increase observed under low confining pressure. The reductions ranged from 10% to 30%. This observed behavior aligns with the fact that biopolymers differ from chemical additives, which induce long-term pozzolanic reactions that develop with extended curing periods [21,59]. The following section investigates the effect of dehydration on the development of resilience behavior under cyclic traffic loading for biopolymer-treated specimens.

### 4.4. Effect of Dehydration DC on M_R_ Values

**Figure 11:**
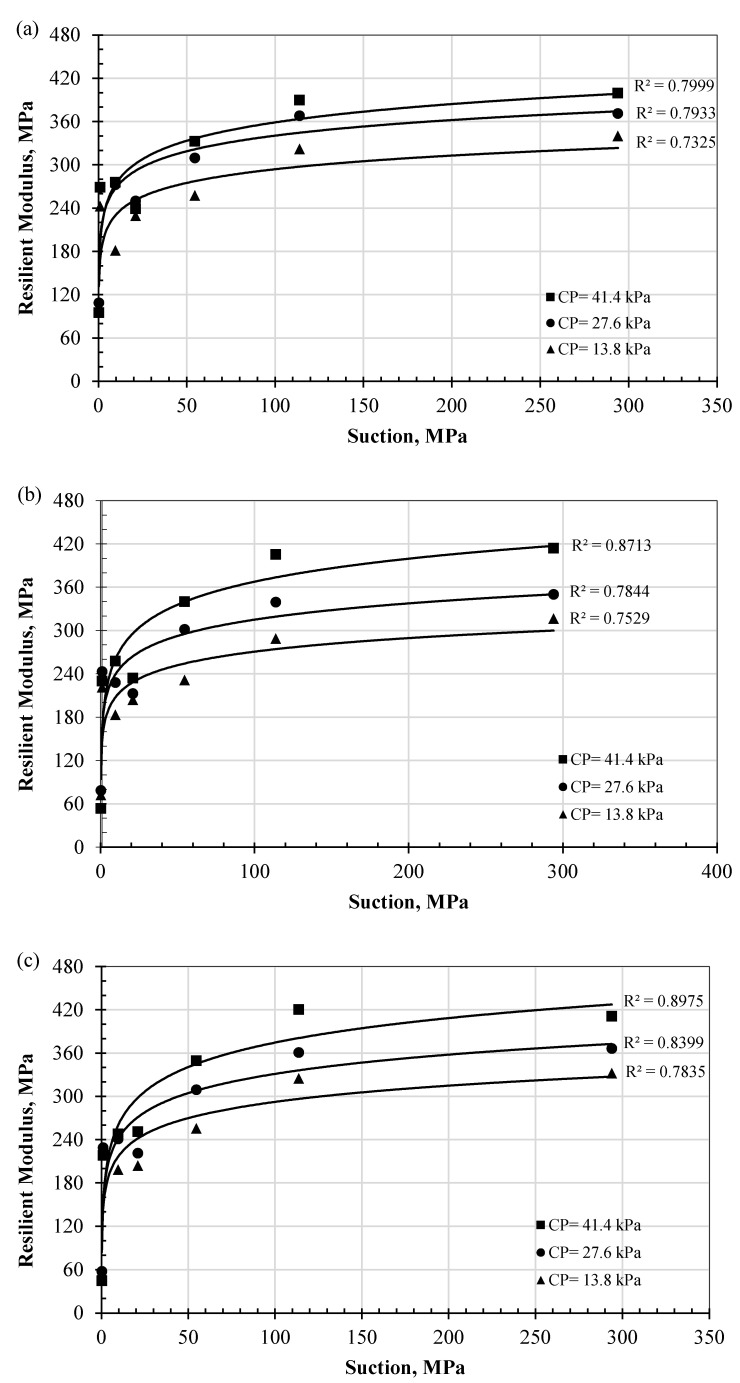
Effect of dehydration on the M_R_ of biopolymer-treated sand at maximum axial stresses of (**a**) 13.8 kPa, (**b**) 41.4 kPa, and (**c**) 68.9 kPa.

The effect of the dehydration process on the M_R_ of the biopolymer-treated soil is presented in this section, and the hydration levels are identified by the suction level, as described earlier (Figure 8). Figure 11a–c present the M_R_ values versus the suction level on the horizontal axis for maximum axial stresses of 13.8, 41.4, and 68.9 kPa, respectively. The results indicate significant development in the M_R_ values with increasing suction level from 0.25 to 1.0 MPa: the value increased by a factor of approximately 3.5 times. This ratio reached nearly eightfold at the highest suction level (294 MPa). This increase could be attributed to the interaction mechanism between soil particles and biopolymers, including coating of soil particles, bridging of interparticle gaps, agglomeration, and hydrogel formation, all of which enhanced cementation between soil particles.

**Figure 12:**
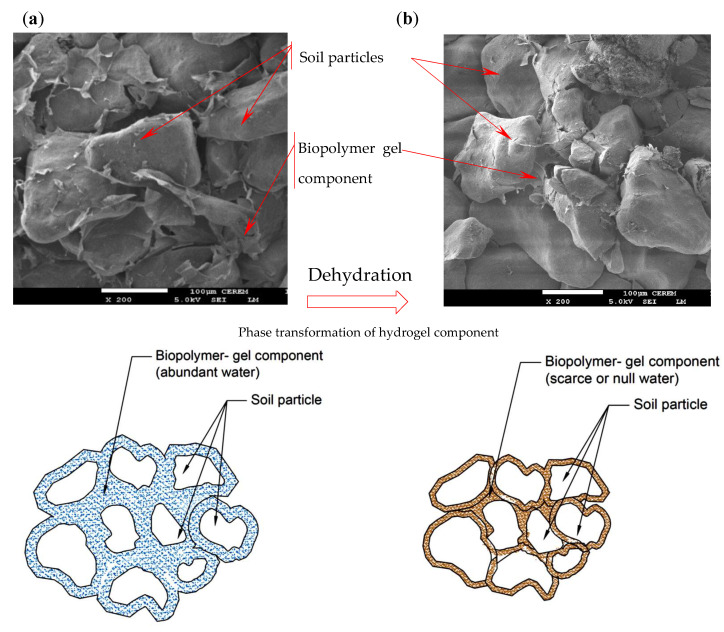
SEM images and interaction mechanism for biopolymer-treated soil: (**a**) before dehydration; (**b**) after dehydration.

In addition, the cross-linking phenomena of biopolymer hydrogels involve both physical and chemical methods, with covalent bonds stabilizing the network structure and enhancing the mechanical behavior and bonding of biopolymer-amended soils [60,61]. These new bonds play a significant role in improving the cohesion and strength of treated soils [15,62,63,64]. For example, improvement has reached 30% in the shear strength in some studies [21]. Furthermore, the dehydration of the specimens enhanced the cross-linking mechanism and hardening of the biopolymer gel. These products coated the soil particles and induced tightened bonds between them. This claim is supported by the SEM images of the treated specimens before and after dehydration, as shown in Figure 12a,b. The associated schematic diagram also illustrates strengthened gel films surrounding the soil particles (Figure 12b). Before dehydration in the presence of water, the hydrogel films were swollen, which may have induced less cementitious bonding. In some cases, these new bonds strengthen soils by a factor of 40 [21,65]. Correlation with different dehydration regimes (SWRC zones) indicates that this mechanism intensifies with ongoing dehydration; thus, increases in suction during dehydration within the transition zones significantly contributed to the increase in M_R_ values. At low water content, where water molecules are tightly bound to soil particles, the biopolymer provided strong interparticle bonding, as indicated in Figure 12b [30]. The capillary zone of this soil remained largely saturated, and no notable dehydration occurred in this zone. At the residual state, M_R_ values increased up to eightfold and then stabilized. In this zone, the dehydration was minimal, and no further internal structural changes took place. At the microstructural level, under conditions of low water attraction (i.e., high suction), cementitious material was formed as a result of chemical interactions between the charges of clay particles.

These findings demonstrate that dehydration is crucial for biopolymer-treated soils. At high suction (low water content), the biopolymer components strengthen interparticle bonding. The phase change from hydrogel to film generates tensile strength, which in turn enhances the interface bonding and resilience characteristics. In contrast, at low suction (high moisture content), the swollen hydrogel weakens bonding among particle networks, reducing the soil’s resistance and resilience to external loads.

From these data, the prediction model can be formulated to predict the M_R_ for biopolymer-treated sandy soil at any level of dehydration or suction at maximum axial stresses of 13.8, 41.4, and 68.9 kPa, as shown in Equations (2), (3), and (4), respectively. The driven model exhibited a good-to-excellent correlation (R^2^) with the measured data, as shown in Figure 9. Further validation in other sandy soils is highly recommended for future studies.

The M_R_ is considered a highly sensitive input parameter in the *Mechanistic–Empirical Pavement Design Guide* (MEPDG), as variations in the M_R_ value can significantly impact the predicted pavement performance and required thickness. Previous studies have reported that improvements in M_R_ can reduce the thickness of pavement layers (i.e., [28,66]).

(2) $${M_{R},}_{\left(MAX:13.8\right)}=\left\{\begin{array}{c} 48.93\ln\left(\psi \right)+149.29;CP=41.4\;\mathrm{kPa}\\ 38.39\ln\left(\psi \right)+154.43;\;CP=27.6\;\mathrm{kPa}\\ 32.419\ln\left(\psi \right)+143.11;\;CP=13.8\;\mathrm{kPa} \end{array}\right.$$

(3) $${M_{R},}_{\left(MAX:41.4\right)}=\left\{\begin{array}{c} 46.001\ln\left(\psi \right)+155.84;CP=41.4\;\mathrm{kPa}\\ 32.527\ln\left(\psi \right)+165.26;\;CP=27.6\;\mathrm{kPa}\\ 25.09\ln\left(\psi \right)+145.93;\;CP=13.8\;\mathrm{kPa} \end{array}\right.$$

(4) $${M_{R},}_{\left(MAX:68.9\right)}=\left\{\begin{array}{c} 36.706\ln\left(\psi \right)+189.8;CP=41.4\;\mathrm{kPa}\\ 31.252\ln\left(\psi \right)+196.55;\;CP=27.6\;\mathrm{kPa}\\ 27.328\ln\left(\psi \right)+168.07;\;CP=13.8\;\mathrm{kPa} \end{array}\right.$$

In the above equations, *M*_R_ indicates the resilient modulus in MPa and *Ψ* indicates the suction in MPa.

## 5. Summary and Conclusions

This study focused on evaluating the effect of dehydration on the resilient modulus (M_R_) of biopolymer-treated sand. Two different curing methods—closed-system curing (CSC) and dehydration curing (DH)—were performed before the M_R_ tests. The soil water retention curve (SWRC) was measured in the laboratory over the entire suction range to design the dehydration curing process, interpret the results in light of the different dehydration regimes, and appropriately model the results with respect to the levels of suction. M_R_ tests were conducted using a dynamic triaxial system, stimulating different field conditions of confining stresses and dynamic traffic loads. The main conclusions are as follows.

- The response of biopolymer-treated sand and its resilience characteristics under dynamic loads were significantly affected by the dehydration regime.
- The SWRC for treated soil exhibited a bimodal shape and defined the different dehydration regimes along the entire range of suction. The major extent of dehydration occurred in the transition regime.
- CSC curing generally reduced M_R_ by approximately 10% to 30%. In the presence of water, strain-softening behavior developed, weakening the resilience characteristics.
- DC resulted in a substantial increase in MR, which rose nearly eightfold up to the residual regime. The most significant development in MR during DC was attained in the transition zone, where dehydration was most active.
- At the highest level of dehydration (high suction), the biopolymer gel components strengthened interparticle bonding. The phase change from hydrogel to films was supported by SEM images, and the enhancement of the cross-linking phenomenon generated tensile strength and improved resilience characteristics.
- Considering the advantages of linking DC and the results of M_R_ to different dehydration regimes of SWRC, a new model was developed to predict M_R_ values, considering the confinement, dynamic axial stress, and suction level.

## Figures and Tables

**Table 1:** Salt solution with corresponding suction.

Salt Solution	Nominal Suction Values, kPa	Measured Suction Values (Using WP4C), kPa	Relative Humidity %
Potassium carbonate (K_2_CO_3_)	113,700	113,910	43.16 ± 0.39
Potassium iodide (KI)	50,376	51,930	68.86 ± 0.24
Potassium chloride (KCl)	21,848	23,290	85.06 ± 0.38
Potassium nitrate (KNO_3_)	8959	9660	93.58 ± 0.55
Sodium chloride (NaCl) *			
NaCl (16.266 gm)/1000 mL water	1000	1070	98.6 ± 0.50
Air-drying	-	294,300	-

* Prepared as per ASTM D 5298 [51].

## Data Availability

The data used to support the findings of this study are included in the figures.
